# Pleasure before business: emotions and age effects on daily activity scheduling

**DOI:** 10.1038/s41598-023-44245-w

**Published:** 2023-10-24

**Authors:** Pasquale La Malva, Irene Ceccato, Adolfo Di Crosta, Nico D’Angelo, Anna Marin, Emanuela Bartolini, Eleonora D’Intino, Nicola Mammarella, Riccardo Palumbo, Rocco Palumbo, Alberto Di Domenico

**Affiliations:** 1 Department of Psychological, Health and Territorial Sciences, “G. d’Annunzio” University of Chieti-Pescara, 31, Via Dei Vestini, 66100 Chieti, Italy; 2 Center for Translational Cognitive Neuroscience, Boston University, VA Boston Healthcare System, Boston, MA USA; 3 Department of Neurology, Boston University School of Medicine, Boston, MA USA; 4 Department of Neurosciences, Imaging and Clinical Sciences, “G. d’Annunzio” University of Chieti-Pescara, Chieti, Italy; 5 Center for Advanced Studies and Technology (CAST), “G. d’Annunzio” University of Chieti-Pescara, Chieti, Italy

**Keywords:** Human behaviour, Psychology

## Abstract

Activity scheduling represents a key process in daily life, involving the evaluation of the costs and benefits of the resources to be invested, but also a preference for when to engage in pleasant or unpleasant activities. Aging affects the evaluation processes and individual preferences due to changes in cognitive functioning and life perspectives. The present study investigated the differences between younger adults (age range 19–33) and older adults (age range 65–87) in a task assessing preferential scheduling of activities. Participants were asked to schedule thirty emotional activities (positive, negative, or neutral) within a 30-day time window, assigning one activity for each day. Results indicated that older adults, but not younger adults, planned positive activities closer in time, supporting the “positivity effect”. Also, both younger and older adults tended to postpone negative activities, scheduling them in the last days. Finally, for older adults only, negative affect was associated with the tendency to put positive activities closer in time. Present findings showed age-specific effects of emotional content on daily activity scheduling.

## Introduction

Choosing what activities to do in the future is a daily process. The scheduling process usually involves an evaluation of the costs and benefits of the resources to be invested, in terms of energy and time. Effective scheduling, in fact, includes a priority analysis of the activities to be carried out to achieve specific objectives^1^. However, the selection of activities also depends on individual needs and subjective preferences on how to invest the time, that is, preferences on when to engage in pleasant, unpleasant, or neutral activities. Evaluations and preferences may change with aging, both for changes in cognitive functions and for variations in life perspectives and expectancies^2^. Indeed, older adults are more motivated to collect and accumulate positive experiences, preferring activities and relationships that can ensure an improvement in mood and well-being in the present time^3^.

Crucially, a large body of studies has shown that time perspective and time processing change with aging^4^. Older adults perceive time’s flow faster than younger adults, resulting in lower accuracy in time judgment tasks^5^. Furthermore, temporal source memory of older adults is significantly impaired, causing more errors in the temporal positioning of past events compared to younger adults^6,7^. Older adults also showed compromised future thinking ability, mirroring the difficulties experienced in the temporal organization of past experiences and memories^8^.

An influential account explaining the age-related changes in time perspective is the socioemotional selectivity theory (SST). According to this theory, older adults perceive the time horizon as more reduced and constrained compared to younger adults^9^. This change in time perspectives influences personal goals, and older individuals are more motivated to collect and accumulate positive experiences, investing resources mainly in activities and relationships that can ensure an improvement in mood and well-being in the present time. According to the SST, this propensity acts as an emotion regulation strategy. Moreover, extensive evidence supports the idea of a preference for positive events, objects, and memories in aging^10–12^. This attitude is known as the “positivity effect” and has been observed in memory, perception, and attention tasks^13–16^. This positivity bias appears to emerge with aging as an evolutionary compensatory effect, serving as a further emotion regulation strategy in approaching the end of one’s life. Indeed, throughout the entire lifespan preceding aging, a negativity bias appears to be prevalent. This phenomenon with opposite valence is known as the “negativity effect” and reflects children’s and younger adults’ enhanced focus on negative stimuli^17,18^. Several studies have investigated the negativity effect, demonstrating how the processing of negative stimuli requires greater cognitive effort in terms of attention and perception compared to positive and neutral stimuli. As a consequence, the negative stimuli are more stably encoded in memory^19,20^. This effect can also have a significant impact on individuals’ decision-making. For the Prospect Theory, the negativity bias may be link to the phenomenon of “loss aversion”^21^. Specifically, when individuals face a choice involving both gains and losses, losses (negative stimuli) are more salient in the decision-making process, receiving greater importance than gains (positive stimuli)^22^. This effect seems to provide an evolutionary advantage, whereby the increased elaboration and storage of negative information contributes to greater survival and enables the avoidance or better coping the same negative experiences in the future^23–25^.

In daily life, all the activities that people schedule have emotional valence, since they are spontaneously classified as more or less pleasant, unpleasant or neutral. In studies of emotions, stimuli can be emotionally classified through a categorical model and a dimensional model. The categorical model involves the specific attribution of the type of emotion elicited by the stimulus, such as happiness, anger, sadness, surprise, disgust, and fear^26^. The dimensional model, or circumplex model, involves evaluating the valence dimension (negative, neutral, and positive) and arousal dimension (calm, neutral, and exciting) of a stimulus. In this study, we classified scheduling activities based on the valence dimension, ranging from negative to positive, with neutral in the midpoint^27^. This affective connotation could influence the assignment of priorities during the scheduling process. Research indicated that younger adults often choose negative stimuli as the first and keep positive stimuli at the end of a sequence, showing a sort of “save the best for the last” tendency^28,29^. Regarding older adults, evidence is sparse and far from conclusive^30^. For instance, Löckenhoff and colleagues^31^ found that in a sequence-preference task, older adults preferred to see negative pictures lastly, compared to pictures with positive and neutral valence. However, no age-related differences emerged when presented with physical and cognitive efforts sequence-preferences tasks^32,33^. In these studies, both younger and older participants selected an improving sequencing, in which the better outcomes (i.e., easier tasks) were saved for the end.

Starting from these premises, the present study aimed to examine differences between younger and older adults in time preferences for future activities as a function of activities’ emotional valence. We created an ecological scheduling task reproducing a real-life context to avoid the potential confounding effects of motivational aspects and task complexity on older adults’ performances. Hence, the selected programmable activities represented common activities in everyday life, with positive, negative, and neutral valence, such as opening a gift and paying a fine. People were requested to plan emotional activities within a 30-day time frame, assigning one activity for each day. This experimental paradigm differed from those previously implemented as it was both hypothetical (i.e., people just thought about activities, without experiencing them) and with a longer time frame (compared to few minutes/hours used in past studies).

Based on the literature, we formulated two main hypotheses: (1) older adults would assign positive activities at the beginning of the schedule and move toward the end of the schedule the negative activities; (2) younger adults would plan negative activities at the beginning of the schedule, and positive activities toward the end of the schedule. In other words, we expected to find opposite trends in the two age groups in scheduling emotional activities, with older adults choosing mainly positive activities and younger adults selecting mostly negative activities, in the first positions of the calendar. As the participants’ emotional state may influence the choices, we also measured the participants’ affectivity (positive and negative). We speculated that individuals’ mood would be related to the order of activities planned, but we did not hypothesize any specific direction for this effect, given the lack of previous studies.

## Results

A Scheduling Index (SI) was created to analyse activities’ positioning preferences (see “Emotional Scheduling Task” section in Methods for more details). Repeated measures ANOVA was performed on SI scores to assess age-related differences in scheduling as a function of activities’ emotional valence. Age group (younger vs. older) was the between-subject factor, and Valence (positive, neutral, vs. negative) was the within-subject factor. The SI scores did not significantly deviate from a normal distribution, Shapiro–Wilk tests, *p*s ≥ 0.109, and the assumption of homogeneity of variance was met, as indicated by Levene’s test, *p*s ≥ 0.409.

**Figure 1:**
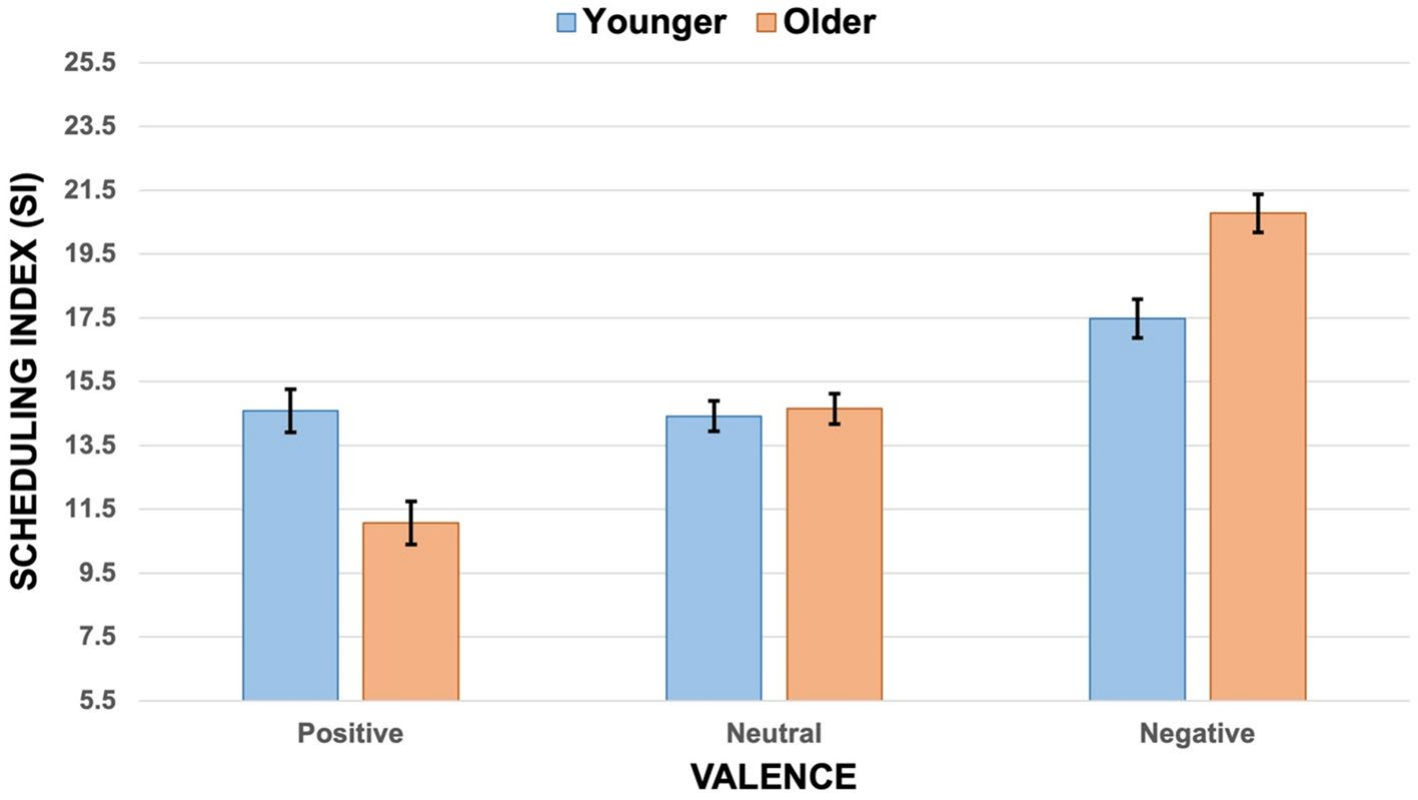
Analysis chart: scheduling index for activities as a function of valence and age group (vertical bars denote 95% confidence intervals).

Following pairwise comparisons (Bonferroni adjusted) indicated a main effect of valence, *F*(2, 100) = 40.37, *p* < 0.001, partial η^2^ = 0.45 (Fig. 1; see Table 1 for means and standard deviations). Results revealed that positive and neutral activities were planned closer in time compared to negative activities, *p*s < 0.001. Also, the Positive SI was smaller than the Neutral SI, yet this difference was not statistically significant, *p* = 0.061.

**Table 1:** Emotional Scheduling Task Results (SI = Scheduling Index): means (*M*) and standard deviations (*SD*) separated by age group.

	Younger		Older		Full sample	
	M	SD	M	SD	M	SD
Positive SI	14.59	0.67	11.07	0.67	12.83	3.84
Neutral SI	14.42	0.48	14.65	0.48	14.53	2.44
Negative SI	17.48	0.60	20.78	0.60	19.13	3.48

Crucially, a significant interaction effect Age group x Valence emerged, *F*(2, 100) = 11.08, *p* < 0.001, partial η^2^ = 0.18. Following Bonferroni adjusted pairwise comparisons showed that, for both younger and older adults, negative activities were planned more distant in time compared to neutral activities, *p* = 0.003 and *p* < 0.001, respectively. However, only for older adults, the Negative SI was greater than the Positive SI, *p* < 0.001, and the Neutral and Positive SI significantly differed, *p* = 0.002.

Inspecting age-related differences, we found that older and younger adults did not differ in Neutral SI, *p* = 0.745. However, older adults showed smaller Positive SI (*p* < 0.001) and greater Negative SI (*p* < 0.001) compared to younger adults. This indicates that older adults tended to plan positive activities first, and negative activities later, to a greater extent than younger adults.

Finally, we examined whether affect was related to activity scheduling. Preliminary analyses indicated that both age groups scored high on the PA scale (younger, *M* = 31.88, *SD* = 3.00; older, *M* = 32.42, *SD* = 6.51) and low on the NA scale (younger, *M* = 19.15, *SD* = 5.60; older, *M* = 18.69, *SD* = 5.72). The two age groups did not significantly differ in affect, neither in positive, *t*(50) = 0.38, *p* = 0.703, nor negative, *t*(50) = 0.29, *p* = 0.770. We conducted correlation analyses separating the two age groups. To control for multiple comparisons, we adjusted *p* values with the Benjamini–Hochberg correction^34^, setting the false discovery rate to 0.05. Results indicated that affect, both positive and negative, was not related to younger adults’ emotional scheduling preferences, adjusted *p*s ≥ 0.238. Interestingly, older adults with higher negative affect planned positive activities closer in time, as reflected by their lower Positive SI, adjusted *p* = 0.049 (Table 2).

**Table 2:** Correlation analyses between SI scores and affect, measured with the PANAS questionnaire.

	Younger		Older	
	PA	NA	PA	NA
Positive SI				
* r*	0.15	0.34	0.10	**− 0.54**
* p*	0.451	0.093	0.640	**0.004**
B-H adjusted *p*	0.577	0.238	0.699	**0.049**
Neutral SI				
* r*	0.24	− 0.27	− 0.15	0.33
* p*	0.233	0.191	0.481	0.097
B-H adjusted *p*	0.399	0.382	0.577	0.238
Negative SI				
* r*	− 0.35	− 0.17	0.02	0.33
* p*	0.083	0.398	0.937	0.099
B-H adjusted *p*	0.238	0.577	0.937	0.238

PA positive affect, NA negative affect.

Pearson’s r, associated p values, and p values adjusted with Benjamini–Hochberg correction are reported.

Significant values are in bold.

## Discussion

This study examined age-related differences in emotional activity scheduling. In line with the SST, results indicated that older adults planned positive activities closer in time than neutral activities, revealing the presence of a “positivity effect”^16^. Extending the SST to scheduling preferences, current results suggested that the constrained time horizon motivates older people to grab positive experiences immediately, scheduling pleasant activities sooner in time.

Recently, Ceccato and colleagues^6^ showed a similar age-related preference using a temporal positioning task of emotional stimuli previously encoded. The authors observed that older adults put positive stimuli closer in time (in the near past) and pushed away (in the far past) negative stimuli, compared to younger adults. Here we found similar evidence for the avoidance of negative stimuli, as older adults planned activities with negative emotional valence more distant in time than activities with neutral valence. This result is also consistent with the studies by Löckenhoff and colleagues^31^, in which older adults tended to postpone negative pictures toward the end of a sequence in a temporal discounting task, even at the cost of viewing more negative images overall.

On the other side, we hypothesized that younger adults would plan negative activities at the beginning of the schedule, and positive activities toward the end of the schedule. The literature showed that younger adults tend to prioritize negative stimuli and experiences, showing a preference for improving sequences^17^. This negative time preference may represent an evolutionary advantage, allowing people to immediately face and resolve a problematic situation and to safely move to other activities^35^. However, we found no evidence of such an effect. Instead, younger adults planned negative activities more distant than neutral activities, as older adults did. We speculate that this could be due to the type and number of stimuli we used in the study. In fact, the negative activities we asked to plan had a low level of dangerousness, rather evoking fatigue and annoyance. Consequently, the evolutionary mechanism was not triggered, and younger adults postponed negative stimuli. Furthermore, the preference for improving sequences has been found when the number of outcomes was limited in size, potentially requesting less effort to select the sequence. Studies requesting to order a greater number of stimuli/outcomes showed a less consistent pattern of results^28,29^.

Notably, in our study, older adults showed a stronger tendency to postpone negative activities compared to younger adults. It is possible that for both the age groups, negative activities, beyond unpleasantness, had an intrinsic tiring nature -they are both physically and cognitively expensive- and thus avoided. However, the negative valence of the stimuli had a greater impact on older adults compared to younger adults. Based on the Selective Engagement Theory, due to their limited cognitive resources, older adults tend to actively select where to allocate attention and energy^36^. Hence, they may be further motivated to avoid effortful activities. Overall, our findings indicated that irrespective of age, people tend to postpone activities with a negative emotional valence, but this effect got more robust with age.

Finally, results suggested that older adults, but not younger adults, may use scheduling as an emotion regulation strategy^37^. Correlation analysis showed that higher negative affect in this age group was associated with lower SI for positive activities. In other words, older adults with greater negative affect also showed a greater tendency to keep positive stimuli close in time. This may be an effective strategy to cope with negative emotions in daily life. Practically, older people experiencing negative emotions could decide to plan a series of positive events within a short timeframe to restore their mood. In general, past research showed that older adults are more likely than younger adults to engage in emotion regulation activities, actively seeking to preserve positive affect^38^. For example, compared to younger adults, older adults limit their interpersonal interactions to those they are familiar with and care the most about. In this way, they expose themselves to mainly predictable and positive emotional experiences^39^.

In conclusion, the present findings showed that younger and older adults are differently affected by emotions in scheduling daily activities. Older adults showed both a preference for positive activities and an avoidance of negative activities, in line with the SST. Younger adults only showed the tendency to postpone negative activities, but to a small extent compared to older people. Furthermore, we found preliminary evidence that older adults use scheduling future activities as an emotion regulation strategy.

This study has some limitations that should be considered in interpreting the results. First, in examining the effect of aging, we focused on an age range potentially too wide. Past studies highlighted the importance of not considering people over 65 as a homogenous “older adults” population^40^. For this reason, future studies should replicate these analyses by considering, for example, two different samples of older adults aged 65–74 and over 75 because the positivity effect may be more salient with aging^6^. Furthermore, based on the theoretical framework we assumed, we took for granted, but not directly assessed, age-related differences in time perspective. In future works, it may be interesting to evaluate individual differences in the time horizon of the participants, administering tests such as the Future Time Perspective^41^. Finally, we classified stimuli into positive, negative, and neutral activities based on a preliminary rating conducted on younger adults. Even if we feel confident that older adults would classify each activity as younger adults did, we cannot exclude that a given activity could be perceived slightly differently by younger and older adults. Similarly, a more gender-balanced evaluation would be necessary since the rating sample consisted of 81% females, and the activities may have a gender bias in the affective connotation. Therefore, future studies should establish a standard set of emotional activities both to avoid the potential confounding effect of age and gender on the perceived valence of the stimuli and to assess the levels of effort and arousal elicited by the activities.

Notwithstanding these caveats, the study offers intriguing insights into people scheduling preferences, starting to fill a gap in the literature on the interplay between emotions and time. Current results also have practical implications for older adults, as emotions may inadvertently affect how people plan their activities in daily life. To sum up, the common saying “business before pleasure” does not seem to hold in aging.

## Methods

### Participants

The sample was composed of 52 healthy participants divided into two groups: 26 younger adults (women, *n* = 12) and 26 older adults (women, *n* = 15). The group of younger adults was aged between 19 and 33 years (*M* = 25.54, *SD* = 3.39) and had between 13 and 16 years of education (*M* = 14.85, *SD* = 1.49). The group of older adults was aged between 65 and 87 (*M* = 71.96, *SD* = 7.30) and had between 8 and 18 years of education (*M* = 11.46, *SD* = 3.64). Older adults had significantly fewer years of education than younger participants, *t*(50) = 4.39, *p* < 0.001. Furthermore, all participants completed the Beck Depression Inventory-II^42^, a 21-item self-reported questionnaire assessing the severity of depressive symptomatology, to exclude any participants at risk of depression. No participant was excluded, as scores were very low (younger, *M* = 6.65, *SD* = 3.60, range 1–13; older, *M* = 6.38, *SD* = 3.38, range 1–15). Also, no age-related differences emerged, *t*(50) = 0.28, *p* = 0.782. Finally, older adults were screened with the Mini Mental State Examination^43^ to exclude participants with potential cognitive impairments. All the older participants achieved a MMSE score of at least 24 points (*M* = 27.96, *SD* = 1.04, range 26–30).

Participants were Italian volunteers recruited through word-of-mouth and received no compensation for their participation. Before starting the experiment, participants read and signed a written informed consent. The study has been performed in accordance with the principles stated in the Declaration of Helsinki and was approved by the Institutional Review Board of Psychology of the Department of Psychological, Health and Territorial Sciences, “G. d’Annunzio” University of Chieti­Pescara.

### Procedure and materials

Each participant was seated in a quiet room with equal brightness and not exposed to sources of disturbance. First, MMSE (only for older adults), PANAS, and BDI-II were administered in this fixed order. Then, the Emotional Scheduling Task was administered.

### Emotional scheduling task

For the creation of the experimental stimuli, we formulated *ex novo* 55 plannable activities (22 positive, 17 negative, and 16 neutral). A preliminary rating study to assess the emotional valence of these 55 activities was conducted on a sample of 187 students of the “G. d’Annunzio” University of Chieti-Pescara (women,* n* = 152; age: *M* = 20.37, *SD* = 3.77; years of education: *M* = 13.18, *SD* = 0.72). We used the Self-Assessment Manikin^44^, a 9-point Likert scale requesting to indicate emotional valence from 1, very negative, to 9, very positive. A score of around 5 indicates stimuli with neutral valence. Specifically, we adopted the following score ranges to classify the emotional valence of the activities: a mean score of "*M* < 4" for the negative, a mean score of "4 ≤ *M* ≤ 6.5" for the neutral, and a mean score of "*M* > 6.5" for the positive. Based on these score ranges, we selected 30 stimuli: 10 positive (*M* = 8.12, *SD* = 0.29), 10 negative (*M* = 3.38, *SD* = 0.67) and 10 neutral activities (*M* = 5.41, *SD* = 0.60) (see the Supplementary Information online for a list of the activities selected).

The 30 selected activities were written, printed, cut, and plasticized with a size of 4.5 × 4.5 cm. A calendar with a 30-day period was created in the form of a grid, each cell measuring 5 × 5 cm, and was printed on a horizontal sheet of paper (21.00 × 29.70 cm) and plasticized. Finally, pieces of Velcro (1 × 1 cm) were glued both on each day of the calendar and behind each activity, to facilitate the placement of the stimuli on the calendar sheet.

**Figure 2:**
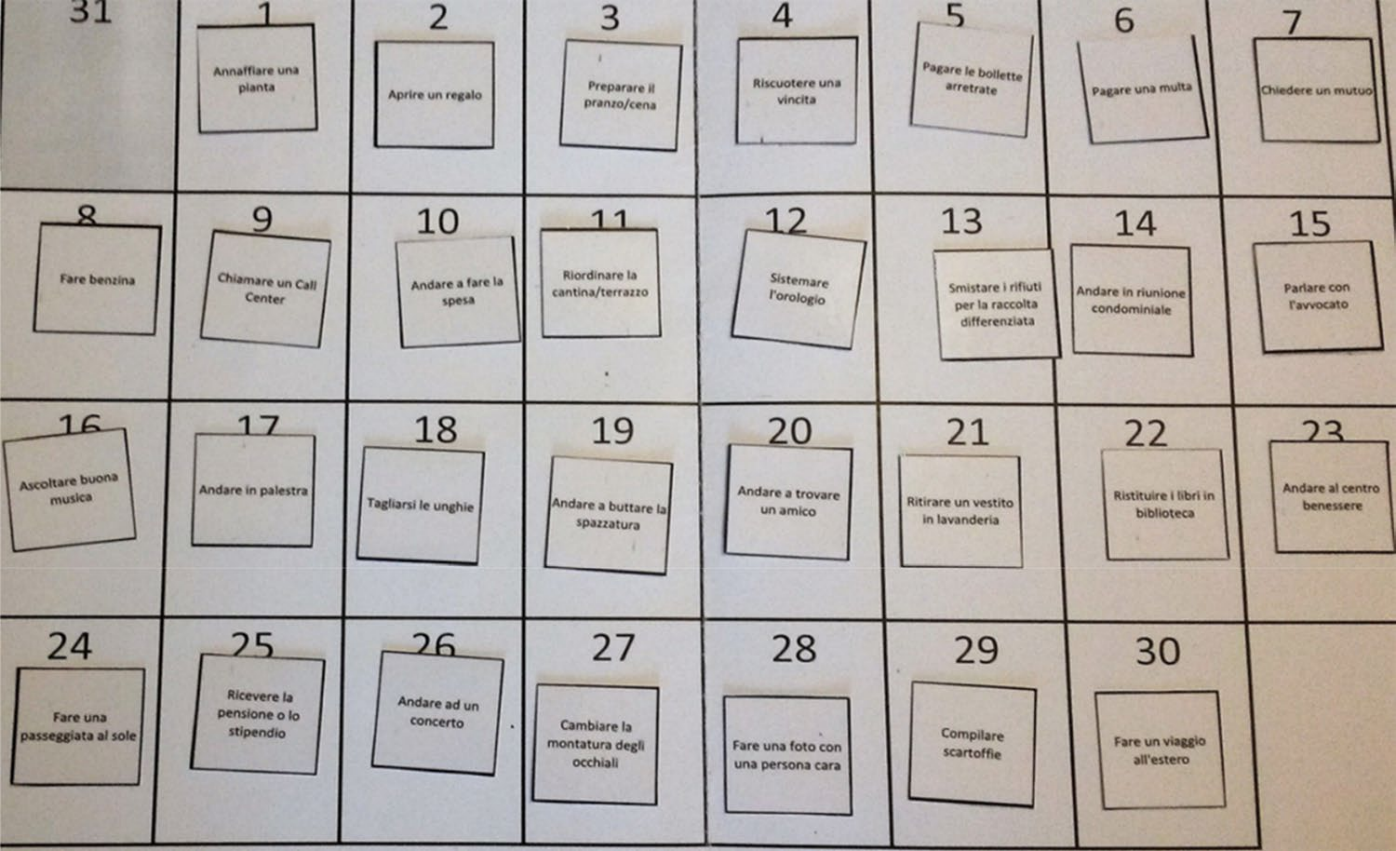
Emotional Scheduling Task: example of a final arrangement of the activities in the calendar.

The task requested participants to place each emotional activity on one of the 30 empty cells of the calendar. The calendar sheet was placed in front of the participant, and the instructions were given orally by the experimenter. Activities were given by the experimenter one at a time in random order. For each day on the calendar, only one activity could be assigned. The participant was allowed to change the order of the activities already entered at any time. When all activities were assigned, and the participant stated to have concluded, the final positioning was recorded and scored later (Fig. 2). Once the task was concluded, the experimenter fully explained the purpose of the study and thanked the participant.

The SI was developed to examine the preferences for the positioning of activities. Three scores were computed, separating positive, negative, and neutral items. Each SI score was computed by averaging the positions (from day 1 to day 30) given to the activities with the same emotional valence. For example, if all the ten positive activities were planned in the first ten days of the calendar, the Positive SI score would be the sum from 1 to 10 (i.e., 55), divided by the number of activities, producing a score of 5.5. Therefore, the closer the index was to the minimum value (5.5), the more the activities with the same valence were distributed in the first days of the month. On the contrary, high SI scores indicated that activities were placed in the final part of the month (the maximum score was 25.5).

### Positive affect and negative affect scales (PANAS)

We evaluated the participants’ affective states by administering the PANAS scale^45^. PANAS is one of the most used questionnaires to evaluate positive and negative affective states and consists of 10 positive adjectives (PA) and 10 negative adjectives (NA). Participants rated how much they felt as described by the adjective during the previous week by responding on a 5-point Likert scale from 1, “not at all”, to 5 “a lot”.

### Supplementary Information

Supplementary Information.

## Data Availability

The dataset and the syntax used to perform the analyses described are freely available at the following link: https://osf.io/56zcx/?view_only=8079ffd1c2eb4ee2bd835c8bb494541d.
