# Supplementary material for: Pleasure before business: emotions and age effects on daily activity scheduling
Source: Sci Rep. 2023 Oct 24;13:18190. doi: 10.1038/s41598-023-44245-w (PMC10598225; doi:10.1038/s41598-023-44245-w)
Supplement: Supplementary file 1 — Supplementary Information. [file 41598_2023_44245_MOESM1_ESM.pdf]

Pleasure before business: Emotions and age effects on daily activity scheduling

Supplementary information

Emotional scheduling task

For the creation of the experimental stimuli, we formulated *ex novo* 55 plannable activities (22 positive, 17 negative and 16 neutral). A preliminary rating study to assess the emotional valence of these 55 activities was conducted on a sample of 187 students of the [anonymised] (F = 152; age: *M* = 20.37, *SD* = 3.77; years of education: *M* = 13.18, *SD* = 0.72). We used the Self-Assessment Manikin (SAM; Bradley & Lang, 1994), a 9-point Likert scale requesting to indicate emotional valence from 1, very negative, to 9, very positive. A score around 5 indicates stimuli with neutral valence. Specifically, we adopted the following score ranges to classify the emotional valence of the activities: a mean score of "$M < 4$" for the negative, a mean score of "$4 \leq M \leq 6.5$" for the neutral, and a mean score of "$M > 6.5$" for the positive.

Based on these ratings, we selected 30 stimuli: 10 positive (*M* = 8.12, *SD* = 0.29), 10 negative (*M* = 3.38, *SD* = 0.67) and 10 neutral activities (*M* = 5.41, *SD* = 0.60). Table S1 presents a list of activities selected.

The 30 selected activities were written, printed, cut, and plasticized with a size of 4.5 x 4.5 cm. A calendar with a 30-days period was created in the form of a grid, each cell measuring 5 x 5 cm and was printed on a horizontal sheet of paper (21.00 x 29.70 cm) and plasticized. Finally, pieces of Velcro (1 x 1 cm) were glued both on each day of the calendar and behind each action, to facilitate the placement of the stimuli on the calendar sheet.

The task requested participants to place each emotional action on one of the 30 empty cells of the calendar. The calendar sheet was placed in front of the participant and the instructions were

given orally by the experimenter. Activities were given by the experimenter one at a time in random order. For each day on the calendar, only one action could be assigned. The participant was allowed to change the order of the activities already entered at any time. When all activities were assigned and the participant stated to have concluded, the final positioning was recorded and scored later. Once the task was concluded, the experimenter fully explained the purpose of the study and thanked the participant.

A scheduling index (SI) was created to analyze activities' positioning preferences. Three scores were computed, separating positive, negative, and neutral items. Each SI score was computed by averaging the positions (from day 1 to day 30) given to the activities with the same emotional valence. For example, if all the ten positive activities were planned in the first ten days of the calendar, the Positive SI score would be the sum from 1 to 10 (i.e., 55), divided by the number of activities, producing a score of 5.5. Therefore, the closer the index was to the minimum value (5.5), and the more the activities with the same valence were distributed among the first days of the month. On the contrary, high SI scores indicated that activities were placed in the final part of the month (with 25.5 as maximum score).

Table S1

List of the action selected following the rating study.

**Action**		**Emotional valence**		
Italian (original)	***English***	***M***	***SD***	***Category***
Fare un viaggio all'estero	Take a trip abroad	8.75	0.71	POS
Andare ad un concerto	Go to a concert	8.37	0.95	POS
Ascoltare buona musica	Listen to good music	8.27	0.92	POS
Ricevere la pensione o lo stipendio	Collect pension or salary	8.20	1.00	POS
Riscuotere una vincita	Collect a win	8.06	1.35	POS
Fare una foto con una persona cara	Take a photo with a loved one	7.98	1.21	POS

Aprire un regalo	Open a gift	7.92	1.18	POS
Fare una passeggiata al sole	Take a walk in the sun	7.89	1.33	POS
Andare al centro benessere	Go to the spa	7.89	1.53	POS
Andare a trovare un amico	Meet with a friend	7.85	1.09	POS
Preparare il pranzo/cena	Make lunch/dinner	6.42	1.73	NEU
Andare in palestra	Go to the gym	6.12	2.24	NEU
Cambiare la montatura degli occhiali	Change the frame of the glasses	5.81	2.14	NEU
Andare a fare la spesa	Go to grocery shopping	5.66	1.63	NEU
Annaffiare una pianta	Water a plant	5.64	1.66	NEU
Restituire i libri in biblioteca	Return the books to the library	5.14	1.62	NEU
Ritirare un vestito in lavanderia	Get a dress from the laundry	5.03	1.45	NEU
Fare benzina	Get gas	4.87	1.61	NEU
Tagliarsi le unghie	Cut the nails	4.80	1.88	NEU
Sistemare l'orologio	Adjust the clock	4.64	1.59	NEU
Smistare i rifiuti per la raccolta differenziata	Sort waste for recycling	3.89	2.04	NEG
Andare in riunione condominiale	Go to a condominium meeting	3.82	1.76	NEG
Riordinare la cantina/terrazzo	Tidy up the basement/terrace	3.78	1.76	NEG
Parlare con l'avvocato	Talk to the lawyer	3.75	1.72	NEG
Chiedere un mutuo	Apply for a mortgage	3.74	1.73	NEG
Andare a buttare la spazzatura	Take out the garbage	3.64	1.86	NEG
Compilare scartoffie	Fill out the paperwork	3.49	1.57	NEG
Chiamare un Call Center	Call a Call Centre	3.36	1.65	NEG
Pagare le bollette arretrate	Pay late bills	2.40	1.58	NEG
Pagare una multa	Pay a fine	1.93	1.22	NEG

Note. In the Emotional valence columns are reported, for each action, the classification, the mean and standard deviation rating of valence, on the SAM scale ranging from 1 to 9 and the resulting classification.

References

Bradley, M. M., & Lang, P. J. (1994). Measuring emotion: The self-assessment manikin and the semantic differential. *Journal of Behavior Therapy and Experimental Psychiatry*, *25*(1), 49–59. https://doi.org/10.1016/0005-7916(94)90063-9
